# Short term effects of milrinone on biomarkers of necrosis, apoptosis, and inflammation in patients with severe heart failure

**DOI:** 10.1186/1479-5876-7-67

**Published:** 2009-07-29

**Authors:** David E Lanfear, Reema Hasan, Ramesh C Gupta, Celeste Williams, Barbara Czerska, Cristina Tita, Rasha Bazari, Hani N Sabbah

**Affiliations:** 1 Department of Internal Medicine, Division of Cardiology, Henry Ford Hospital, Detroit, Michigan, USA; 2 Department of Internal Medicine, Division of Cardiology, Providence Hospital, Southfield, Michigan, USA; 3 Department of Internal Medicine, Division of Cardiology, Beaumont Hospital, Royal Oak, Michigan, USA

## Abstract

**Introduction:**

Inotropes are associated with adverse outcomes in heart failure (HF), raising concern they may accelerate myocardial injury. Whether biomarkers of myocardial necrosis, inflammation and apoptosis change in response to acute milrinone administration is not well established.

**Methods:**

Ten patients with severe HF and reduced cardiac output who were to receive milrinone were studied. Blood samples were taken just before initiation of milrinone and after 24 hours of infusion. Dosing was at the discretion of the patient's attending physician (range 0.25–0.5 mcg/kg/min). Plasma measurements of troponin, myoglobin, N-terminal-pro-BNP, interleukin-6, tumor necrosis factor-α, soluble Fas, and soluble Fas-ligand were performed at both time points.

**Results:**

Troponin was elevated at baseline in all patients (mean 0.1259 ± 0.17 ng/ml), but there was no significant change after 24 hours of milrinone (mean 0.1345 ± 0.16 ng/ml, p = 0.44). There were significant improvements in interleukin-6, tumor necrosis factor-α, soluble Fas, and soluble Fas-ligand (all p < 0.05) indicative of reduced inflammatory and apoptotic signaling compared to baseline.

**Conclusion:**

In conclusion, among patients with severe HF and low cardiac output, ongoing myocardial injury is common, and initiation of milrinone did not result in exacerbation of myocardial injury but instead was associated with salutary effects on other biomarkers.

## Introduction

Intravenous inotropic agents (inotropes) such as dobutamine and milrinone can produce improvements in cardiac output and patient's symptoms via increased contractility and heart rate. However, these type of agents have also been associated increased arrhythmia risk and other adverse outcomes in heart failure (HF) [1-3]. This raises concern that inotropes may cause or contribute to myocardial destruction through worsening ischemia, increased neurohormonal activation, or via other adverse pathways such as inflammation and apoptosis. Biomarkers may provide a glimpse into this pathophysiology without the need for tissue sampling. Modern, high-sensitivity troponin assays can detect even small amounts of myocardial necrosis and natriuretic peptides are well known indicators of cardiac dysfunction and filling pressures. In addition, certain other biomarkers are known to be indicators of inflammation and apoptosis, two processes which accumulating data suggest are important in the pathophysiology of HF.

It is well recognized that heart failure leads to increased circulating levels of pro-inflammatory cytokines, such as tumor necrosis factor α (TNFα) and Interleukin 6 (IL6), which may cause or potentiate progressive cardiovascular injury, [4] and have been associated with increased morbidity and mortality in patients with HF [5]. More recently apoptosis has been investigated as a pathophysiologic mechanism in HF. A key apoptotic signaling system, the Fas/Fas ligand system, shows increased activity in HF patients and correlates to disease severity [6,7]. To briefly summarize, soluble Fas-Ligand (sFas-L) binding to membrane-bound Fas triggers apoptosis, whereas soluble Fas (sFas) competes with membrane-bound Fas for ligand binding, therefore reducing apoptotic signaling.

How these biomarkers change in response to administration of a positive inotropic agent in severe HF is not firmly established. There have been several studies examining natriuretic peptide levels and/or inflammatory markers during inotrope administration with inconsistent results [8-11]. Adding complexity to this picture is data indicating that the specific inotrope used is important as well. For example, there are studies suggesting differences in biomarker effects between dobutamine vs. levosimendan [12], and dobutamine vs. milrinone [13]. Furthermore, there is little or no data regarding the effect of milrinone on apoptosis markers, or whether high-sensitivity troponin may reveal sub-clinical cardiac injury due to inotrope initiation. We sought to determine the effect of initiating milrinone on biomarkers of myocardial function (N-terminal pro-B-type Natriuretic Peptide), myocardial necrosis (troponin I, myoglobin), inflammation (TNFα, IL6) and apoptosis (sFas, sFas-L).

## Methods

### Patients

This study was approved by the Institutional Review Board, and all patients gave written informed consent. Severe heart failure patients undergoing non-urgent right heart catheterization were screened for inclusion from June 2006 to November 2007. After catheterization, patients who were planned by their physician to receive intravenous milrinone due to reduced cardiac output were approached for study participation. A total of 10 patients with NYHA Class IV symptoms and cardiac index <2.0 L/m/M2 were enrolled. After the initial procedure, patients were admitted to the cardiac intensive care with the catheter remaining in place for drug initiation and monitoring as per standard care. Exclusion criteria included exposure to intravenous inotropic support within 1 month and inability to give written informed consent. After conclusion of study participation all patients care continued to be at the discretion of the attending physician, including inotrope administration and dosing.

### Procedures

All treatments including milrinone dosing was at the discretion of the patient's attending physician, with initial dosing between 0.25 and 0.5 μg/kg/min. Patients were observed for at least 24 h. Blood samples were obtained by standard venipuncture from all patients just prior to milrinone initiation (day 0) and after 24 hours of continuous infusion (day 1). Blood samples were centrifuged, plasma aliquoted, and frozen at -70°C until the time of testing. Plasma levels of Troponin I (TnI) and myoglobin (Myo) were measured using sandwich immunoassays with chemiluminescence using the Centaur instrument (Siemens Corporation, Deerfield, Illinois). TnI levels were replicated on each sample to assess precision of measurement, yielding an inter-assay correlation coefficient >0.995. TNFα, sFas, sFas-L and IL6 were determined in plasma using double antibody sandwich Enzyme Linked Immunosorbant Assays (ELISA). NTproBNP level was determined in plasma based on competitive ELISA as described elsewhere [14]. The concentration of each biomarker was assayed using commercially available assay kits according to manufacturer protocol and using standard curves and software. The kits for NTproBNP (fmol/ml) were purchased from ALPCO Diagnostics, Salem, New Hampshire; for IL-6 (pg/ml) and TNFα (pg/ml) from Assay Designs Inc., Ann Arbor, Michigan; and for sFas (pg/ml) and sFas-L (pg/ml) from R&D Systems, Inc, Minneapolis, Minnesota.

### Statistical Analysis

Statistical comparisons were made between baseline levels and 24 hour levels using the paired t-test. P values < 0.05 were considered significant. Power estimate for TnI was 90% to detect a mean difference between time-points as small as 0.02 ng/ml (using experimentally determined correlation coefficient in calculations). All statistics were calculated using SAS 9.1.3. All data are reported as the mean ± standard deviation.

## Results

Baseline characteristics are shown in Table 1. Overall this was a very ill patient cohort with mean ejection fraction of 16%, pulmonary capillary wedge pressure of 30 mmHg and cardiac index of 1.81 L/min/m^2^. TnI and B-type Natriuretic Peptide (BNP) levels were elevated at baseline in all patients (TnI range 0.0205–0.56 ng/ml, mean 0.1259 ± 0.17 ng/ml; mean BNP range 73 to 1620, mean 803 ± 630 pg/ml). On average there was a large improvement in hemodynamics over 24 hours with average cardiac index increasing to 2.5 L/m/M^2^, and mean pulmonary capillary wedge pressure decrease over that period to 23 mmHg.

**Table 1:** Patient Characteristics

**Characteristic**	**Average (± SD)**
Age (yrs)	52 (± 17)

Sex (male/female)	8/2

Ejection Fraction (%)	16% (± 8.18)

Ischemic/Non Ischemic etiology(%)	3 (30%)/7 (70%)

Beta adrenergic antagonist	9 (90%)

Angiotensin converting enzyme inhibitor or angiotensin receptor blocker	3 (30%)

Furosemide	8 (80%)

Furosemide continuous infusion	2 (20%)

Creatinine (mg/dL)	1.73 (± 0.83)

BNP (ng/ml)	803 (± 630)

Pulmonary capillary wedge pressure, baseline (mmHg)	30 (± 8.5)

Pulmonary capillary wedge pressure, @ 24 hours (mmHg)	23 (± 5.0)

Cardiac Index, baseline (L/min/m^2^)	1.81 (± 0.63)

Cardiac Index @ 24 hours (L/min/m^2^)	2.51 (± 0.74)

**Figure 1:**
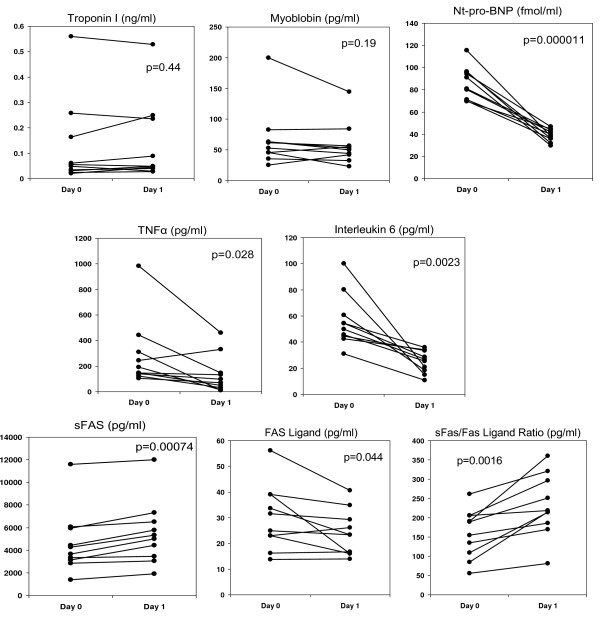
**Biomarker changes from baseline (Day 0) to 24 hours of infusion (Day 1)**.

The change in each biomarker for each participant over the study period is depicted in Figure 1. Compared to baseline, NT-pro BNP levels decreased by 47.5 fmol/ml or 55% (from 86.5 to 39.0 fmol/ml, p < 0.0001). There was no significant change in mean TnI or MYO after 24 hours of milrinone compared to baseline (mean TnI 0.1345 ± 0.16, ↑0.0086 ng/ml or 6.8% compared to baseline, p = 0.44; MYO ↓8.8 ng/ml or 13%, p = 0.19). In contrast there were significant reductions in inflammatory and apoptotic signaling after Milrinone infusion. Levels of IL6 and TNFα were reduced by roughly half after 24 hours of milrinone (IL6 ↓31 pg/ml or 56%, p = 0.0023; TNF↓149 pg/ml or 53%, p = 0.028). In terms of apoptotic signaling, sFas, sFas-L, and the ratio of sFas:sFas-L all changed significantly in a favorable direction over the study period. Soluble Fas levels increased 18% (p = 0.00074) while Fas-Ligand levels decreased 20% (p = 0.044). As a result the sFas:sFas-L ratio increased by 45% (p = 0.0016), consistent with reduced apoptotic signaling. Neither the milrinone dose nor the presence of oral vasodilators were associated with differences in biomarker changes (all p > 0.1).

## Discussion

In this sample of patients with severe HF and reduced cardiac output, initiation of milrinone therapy did not result in changes indicative of accelerated myocardial necrosis, but instead was associated with salutary effects on all the other markers. As might be expected, inotropic support led to improvements in hemodynamic status reflected in increased cardiac output and reduction in NTproBNP levels. Surprisingly, there was no significant change in TnI or MYO after 24 hours of milrinone compared to baseline. On the other hand, there were significant reductions in inflammatory and apoptotic signaling with milrinone infusion. This is the first data we are aware of to show improvements in apoptotic markers with milrinone infusion.

Our findings are notable in several ways. The fact that all of the subjects had measurable TnI at baseline suggests that patients with very advanced HF have ongoing myocardial injury. The lack of worsening of the TnI leak suggests that milrinone does not exacerbate the underlying pathologic process in these patients, at least in the short term. This should be interpreted with caution however, since the majority of the study subjects had a non-ischemic etiology for their HF. This is an especially important factor since patients with ischemic disease seemed to be at greater risk in the OPTIME study [15]. The marked improvements seen in inflammatory and apoptotic markers were somewhat surprising, suggesting a possible benefit of this therapy in properly selected patients. Our patients were extremely ill with low cardiac index and evidence of ongoing myocardial damage as mentioned above. It is possible that in such a state, intervening with inotropes may mitigate the overall neurohormonal activation (including inflammation). If this is the case, it is also possible that this potential benefit may outweigh the possible adverse effects of inotropic agents in the short term.

An additional complexity is that the witnessed effects may not be applicable to all inotropes but instead could be specific to milrinone. For example, milrinone is known to be a more potent vasodilator compared to dobutamine. This relatively enhanced vasodilitation could theoretically account for a more favorable impact on biomarkers. In addition while it is impossible with to completely separate the hemodynamic improvement from other potential effects of milrinone, there is some previous data that reveal differences between inotropic agents in terms of biomarker changes despite similar hemodynamic properties. For example, dobutamine failed to decrease NTproBNP or TNF while levosimendan significantly decreased both in one randomized study [12]. On the other hand, levosimendan infusion decreased sFAS while our data showed a significant increase, suggesting a more favorable effect of milrinone.

Furthermore, previous *in-vitro *data indicates that phosphodiesterase inhibition suppressed TNFα production in mononuclear cells [16,17]. These facts together are consistent with the possibility of a phosphodiesterase-specific effect, perhaps via inflammatory or other pathways, as opposed to a more general inotrope effect based solely on improved hemodynamics.

There are limitations of this study that should be noted. First, the study was non-random and uncontrolled in design. Since inotropic agents are currently considered to carry excess risk and thus are used only when thought to be absolutely clinically necessary, randomization and placebo control was not practical. Another related concern is whether standard therapy, particularly increased loop diuretic dosing, could explain the findings and confound the milrinone effects. In terms of TnI levels, there is no reason to believe that standard therapy would obscure detection of increased myocardial necrosis. While standard care with higher diuretic dosing likely contributed the lowering of NTproBNP levels, it is unlikely to explain the changes seen in the inflammatory and apoptotic markers. Not only has diuretic use been shown to increase neurohormonal activation [18]. but a randomized placebo controlled study of levosimendan in decompensated HF patients revealed that standard therapy including diuretic did not reduce IL6 or TNFα, nor change sFas levels (in contrast to levosimendan) [19]. Other standard therapies such as ACE-inhibitors and beta adrenergic antagonists are very unlikely to be manipulated significantly in this setting due to the severity of the subject's HF. The second main limitation is the small sample size. While the size precludes examination of clinical endpoints, our power estimates indicate that the sample size of 10 was adequate for the planned analyses of biomarkers reported. It is possible that the observation window was too short to observe troponin changes but we feel this is unlikely given that standard 'rule out' of myocardial infarction (necrosis) involves troponin measurements that are typically <12 hours apart. Finally, extrapolation of our results to groups not adequately represented should be avoided. Specifically, these subjects were end-stage patients and mostly of non-ischemic etiology. Consequently, this data does not give as much insight regarding inotrope use in the setting of more routine decompensated heart failure, and the effect milrinone in ischemic subset of patients deserves further investigation.

## Conclusion

Initiation of milrinone therapy in patients with severe heart failure and reduced cardiac output did not result in changes indicative of accelerated myocardial injury. On the contrary, it was associated with significant improvement in biomarkers of the inflammatory and apoptotic pathways. This data does not support the hypothesis that inotrope use is inherently detrimental in all cases, but instead suggests that properly selected patients may have benefits from this treatment, at least in the short-term. Placebo-controlled, randomized studies in patients with low cardiac output are needed to further establish the potential benefits and adverse consequences of the use of positive inotropic agents in this population. Additional studies are also needed to assess longer-term biomarker trends during chronic milrinone infusions and the relationship to clinical outcomes.

## Competing interests

The authors declare that they have no competing interests.

## Authors' contributions

DL conceived of the study, participated in design, coordination, data interpretation, performed the statistical analysis, and drafted the manuscript.

RH participated in design and coordination of the study, data collection, and critically revised the manuscript. RG performed the molecular assays and critically revised the manuscript. CW participated in data collection, interpretation, and critically revised the manuscript. BC participated in data collection, interpretation, and critically revised the manuscript. CT participated in data collection, interpretation, and critically revised the manuscript. RB participated in design and coordination of the study, data collection, and critically revised the manuscript. HS conceived of the study, participated in design, interpretation of data, and critically revised the manuscript. All authors read and approve of the final manuscript.
